# Supplementary material for: H4K20me1 and H3K27me3 are concurrently loaded onto the inactive X chromosome but dispensable for inducing gene silencing
Source: EMBO Rep. 2021 Feb 19;22(3):e51989. doi: 10.15252/embr.202051989 (PMC7926250; doi:10.15252/embr.202051989)
Supplement: Supplementary file 1 — Expanded View Figures PDF [file EMBR-22-e51989-s001.pdf]

Expanded View Figures

Figure EV1. Establishing H3K27me3 mintbody to visualise H3K27me3 in living cells.

A Amino acid sequence of the scFv region of H3K27me3-mintbody derived from clone 2E12. Two amino acids that are substituted from the original 2E12 sequence are indicated with the regions of heavy and light chain variable fragments (V_H and V_L).

B Distribution of mintbody clones. Shown are single confocal sections of living mouse MC12 cells that transiently express mintbodies (sfGFP) from the original 2E12 and single (M86L) and double (M86L M158I) mutants. Scale bar = 10 µm.

C Model structures of 2E12 scFv. The 2E12 scFv structure was generated using ABodyBuilder (Leem *et al*, 2016) and the sites of substituted amino acid residues (coloured in orange) are indicated with the zoomed view. Substitutions from Met86 to Leu and Met158 to Ile can fill the gap (red arrows) and stabilise the hydrophobic cores in heavy (M86L) and light (M158I) chains. Nucleotide sequence of 2E12LI (M86L, M158I) is available under the accession number LC597262 in databases (DDBJ/EMBL/GenBank).

© 2021 The Authors

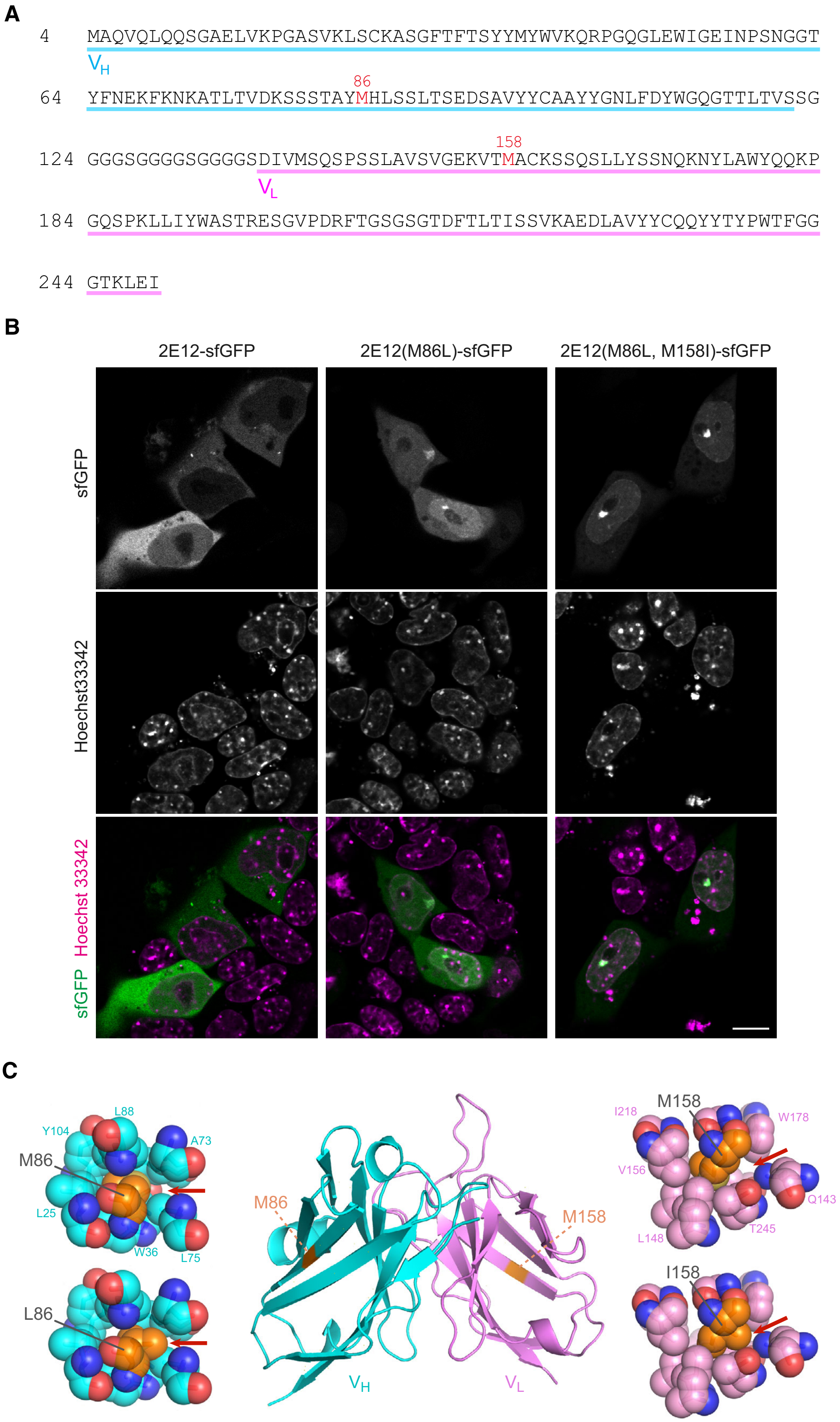
A
4 MAQVQLQQSGAELVKPGASVKLSCKASGFTFTSYYMYWVKQRPGQGLEWIGEINPSNGGT
VH
86
64 YFNEKFKNKATLTVDKSSSTAYMHLSSLTSEDSAVYYCAAYYGNLFDYWGQGTTLTVSSG
158
124 GGGSGGGGSGGGGSDIVMSQSPSSLAVSVGEKVTMACKSSQSLLYSSNQKNYLAWYQQKP
VL
184 GQSPKLLIYWASTRESGVPDRFTGSGSGTDFTLTISSVKAEDLAVYYCQQYYTYPWTFGG
244 GTKLEI
B
2E12-sfGFP
2E12(M86L)-sfGFP
2E12(M86L, M158I)-sfGFP
sfGFP
Hoechst33342
sfGFP Hoechst 33342
C
L88
Y104
A73
M86
L25
W36
L75
L86
M86
M158
VH
VL
I218
M158
W178
V156
Q143
L148
T245
I158

Figure EV1.

Figure EV2. Characterisation of H3K27me3-mintbody.

A SDS-polyacrylamide gel analysis of purified mintbody. MBP-tagged H3K27me3-mintbody was expressed in and purified from *Escherichia coli* through maltose-resin. After the removal of MBP moiety, H3K27me3-mintbody was separated on a 10–20% SDS-polyacrylamide gel and stained with Coomassie Brilliant Blue.

B Peptide array analysis. A MODified Histone Array (Active Motif) was probed with the purified H3K27me3-mintbody followed by peroxidase-conjugated anti-GFP antibody. Peptides containing H3K27me3, except one with H3S28ph, were highlighted. Peptide sequences of highlighted locations were listed in the lower table.

C–F Immunofluorescence. HeLa cells were transfected with expression vectors to transiently express HaloTag-tagged KDM6B (C and E) and KDM4D (D and F), fixed, and stained with antibodies specific for H3K27me3 (C) and H3K9me3 (D), or H3K27me3-mintbody (E and F). Fluorescence intensities in individual nuclei were measured and dot plotted. Transfected cells are indicated by arrowheads. Cells expressing KDM6B and KDM4D showed decreased levels of H3K27me3 (C) and H3K9me3 (D), respectively, by antibody staining. H3K27me3-mintbody signals were lower in cells expressing KDM6B (E) but not KDM4D (F). Scale bars = 10 μm.

 © 2021 The Authors

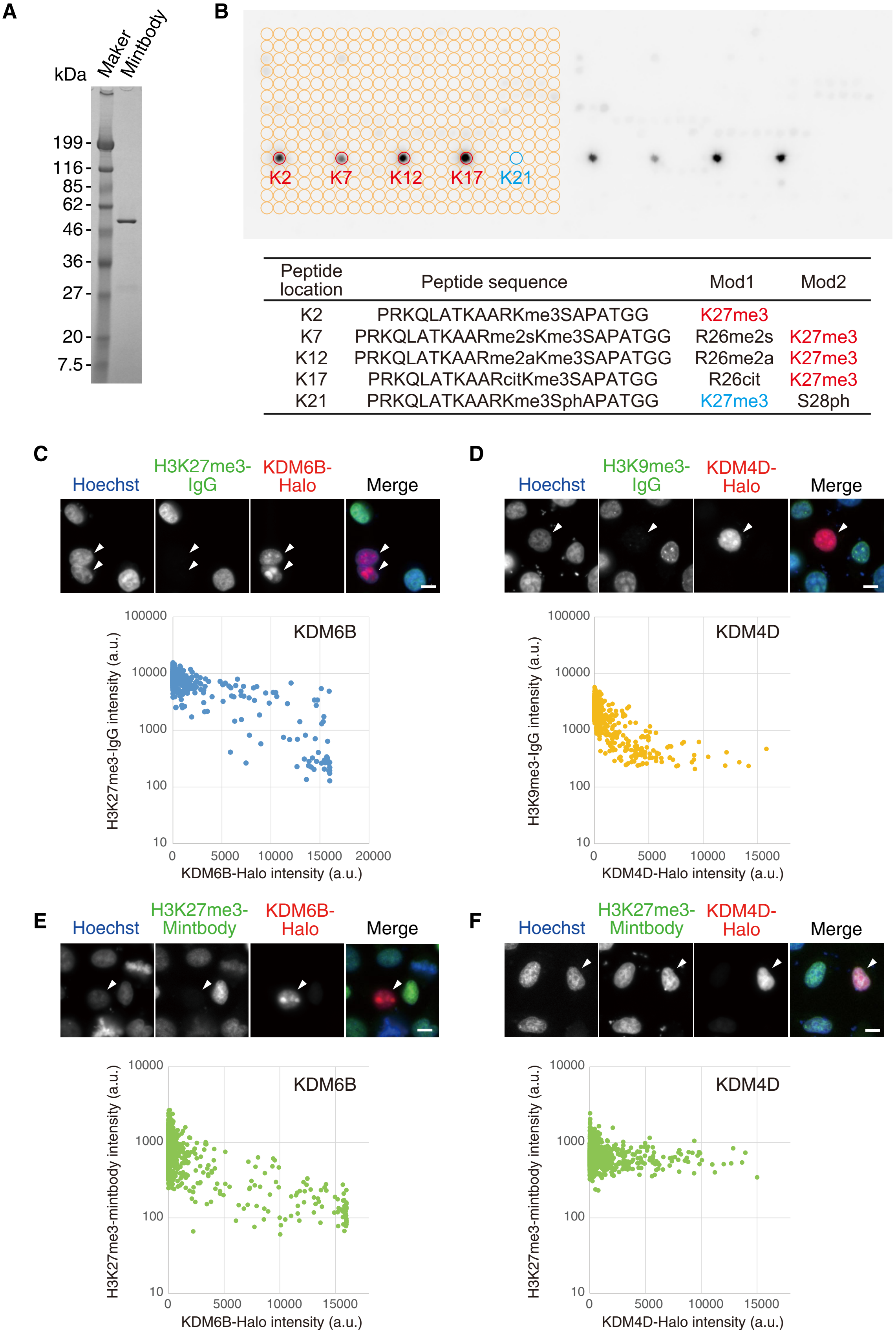

Peptide location	Peptide sequence	Mod1	Mod2
K2	PRKQLATKAARKme3SAPATGG	K27me3	
K7	PRKQLATKAARme2sKme3SAPATGG	R26me2s	K27me3
K12	PRKQLATKAARme2aKme3SAPATGG	R26me2a	K27me3
K17	PRKQLATKAARcitKme3SAPATGG	R26cit	K27me3
K21	PRKQLATKAARKme3SphAPATGG	K27me3	S28ph

Figure EV2.

Figure EV3. Labelling X-linked loci by sgRNA-dCas9 system.

A Schematic diagram of CRISPR/dCas9-3×sfGFP targeting loci on the mouse X chromosome.

B, C IF validation of sgRNAs targeting X-linked loci. Mouse MC12 cells that stably express sgRNA and dCas9-EGFP were fixed and stained with antibody specific for H3K27me3 (Cy5) and Hoechst33342. Shown are cells in interphase (B; maximum intensity projections of 7 z-plane confocal sections) and in mitosis (C; 30 z-sections). Arrowheads and arrow indicate Xa and Xi, respectively. In MC12 cells, the inactive X chromosome is known to have a translocation to an autosome at the distal end and probably lacks mX2 target sequence. Scale bar = 10 μm.

 © 2021 The Authors

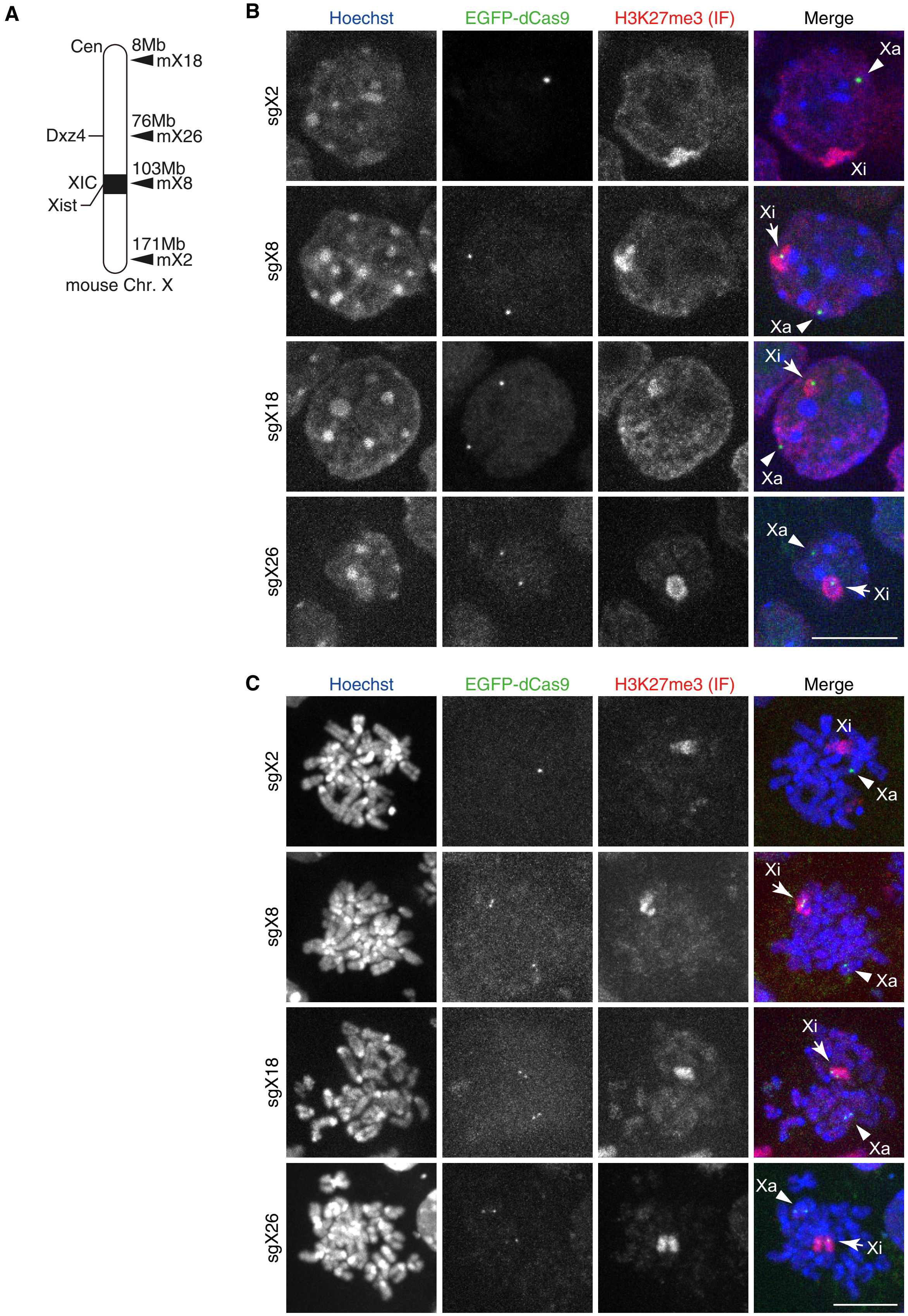
A
Cen
8Mb
mX18
Dxz4
76Mb
mX26
XIC
103Mb
mX8
Xist
171Mb
mX2
mouse Chr. X
B
Hoechst
EGFP-dCas9
H3K27me3 (IF)
Merge
sgX2
sgX8
sgX18
sgX26
Xa
Xi
C
Hoechst
EGFP-dCas9
H3K27me3 (IF)
Merge
sgX2
sgX8
sgX18
sgX26
Xa
Xi

Figure EV3.

Figure EV4. System to simultaneously visualise H3K27me3 or H4K20me1 and *Xist* RNA in living cells.

A Measurement of H4K20me1 (by IF-RNA FISH) and H4K20me2/3 (by IF in TX-*Xist*-mCherry line) enrichment on the Xi. Xist-inducible female cells were treated with DOX for 24 h prior to fixation. Scale bar = 2 µm.

B Schematic representation of the TX-BglSL parental line obtained previously (Dossin *et al*, 2020). This system is based on the TX1072 female hybrid ESC line where *Xist* is induced by DOX treatment from the endogenous B6 allele. In TX-BglSL 18 Bgl stem loops have been knocked-in into the 7th exon of *Xist*.

C Schematic representation of the TX-*Xist*-EGFP; H4K20me1-mCherry ESC line. This line is derived from TX-BglSL by knocking-in BglG-EGFP expression cassette into Rosa 26 locus. Homologous recombination was aided by using nickase Cas9 protein and two gRNAs targeting Rosa 26. Knock-in can occur only at one allele as the other one contains the rTTA element allowing for DOX-inducible expression of *Xist* RNA. Stable lines expressing H4K20me1 specific mintbody fused to mCherry were generated by random insertion of a PiggyBac vector (bottom).

D Schematic representation of the TX-*Xist*-mCherry; H3K27me3-sfGFP ESC line. This line is derived from TX-BglSL by knocking-in BglG-mCherry expression cassette into Tigre locus. Homologous recombination was aided by using wild-type Cas9 protein and a single gRNA targeting Tigre. Stable lines expressing H3K27me3 specific mintbody fused to sfGFP were generated by random insertion of a PiggyBac vector (bottom).

E Validation of efficient X chromosome inactivation in TX-*Xist*-EGFP; H4K20me1-mCherry and TX-*Xist*-mCherry; H3K27me3-sfGFP ESC lines. Cells were treated with DOX for 0 or 24 h; pyrosequencing was performed from cDNAs. Allelic ratios of two X-linked genes (*Rnf12* and *AtrX*) were measured. Note comparable levels of gene silencing in both clones.

F Quantification of *Xist* induction efficiency during live imaging analysis upon DOX treatment. Percentage *Xist*-Bgl-EGFP clouds was manually assessed in at least 30 cells.

© 2021 The Authors

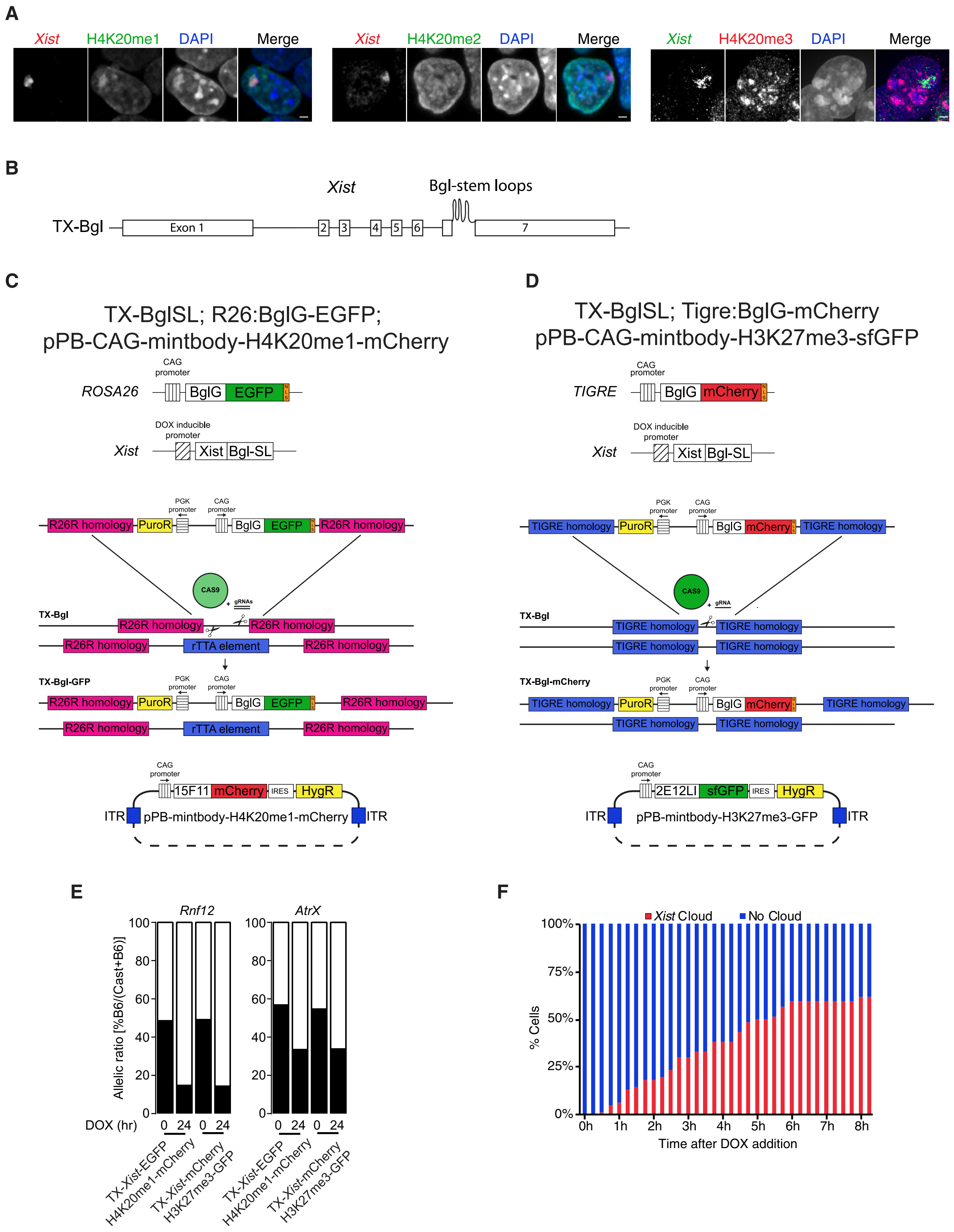
A
Xist
H4K20me1
DAPI
Merge
Xist
H4K20me2
DAPI
Merge
Xist
H4K20me3
DAPI
Merge
B
Xist
Bgl-stem loops
TX-Bgl
Exon 1
C
TX-BglSL; R26:BglG-EGFP;
pPB-CAG-mintbody-H4K20me1-mCherry
ROSA26
Xist
D
TX-BglSL; Tigre:BglG-mCherry
pPB-CAG-mintbody-H3K27me3-sfGFP
TIGRE
Xist
E
Rnf12
AtrX
Allelic ratio [%B6/(Cast+B6)]
DOX (hr)
TX-Xist-EGFP
H4K20me1-mCherry
TX-Xist-mCherry
H3K27me3-GFP
F
Xist Cloud
No Cloud
% Cells
Time after DOX addition

Figure EV4.

A

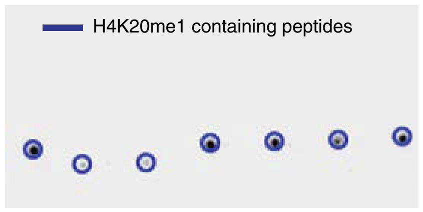

Peptide location	Peptide sequence	Mod1	Mod2	Mod3
N 1	G K G G A K R H R Kme1 V L R D N I Q G I T	**K20me1**		
N12	G K G G A Kac R H R Kme1 V L R D N I Q G I T	K16ac	**K20me1**	
N16	G Kac G G A Kac R H R Kme1 V L R D N I Q G I T	K12ac	K16ac	**K20me1**
N20	G K G G A K R H Rme2a Kme1 V L R D N I Q G I T	R19me2a	**K20me1**	
N24	G K G G A K R H Rme2s Kme1 V L R D N I Q G I T	R19me2s	**K20me1**	
O 4	G K G G A K R H R Kme1 V L Rme2a D N I Q G I T	R24me2a	**K20me1**	
O 8	G K G G A K R H R Kme1 V L Rme2s D N I Q G I T	R24me2s	**K20me1**	

B

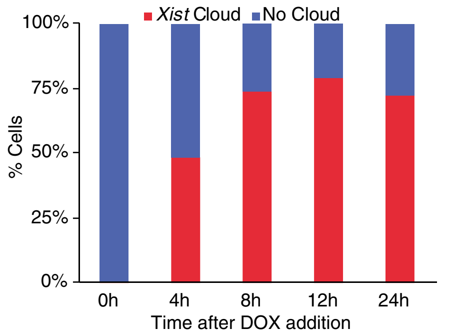

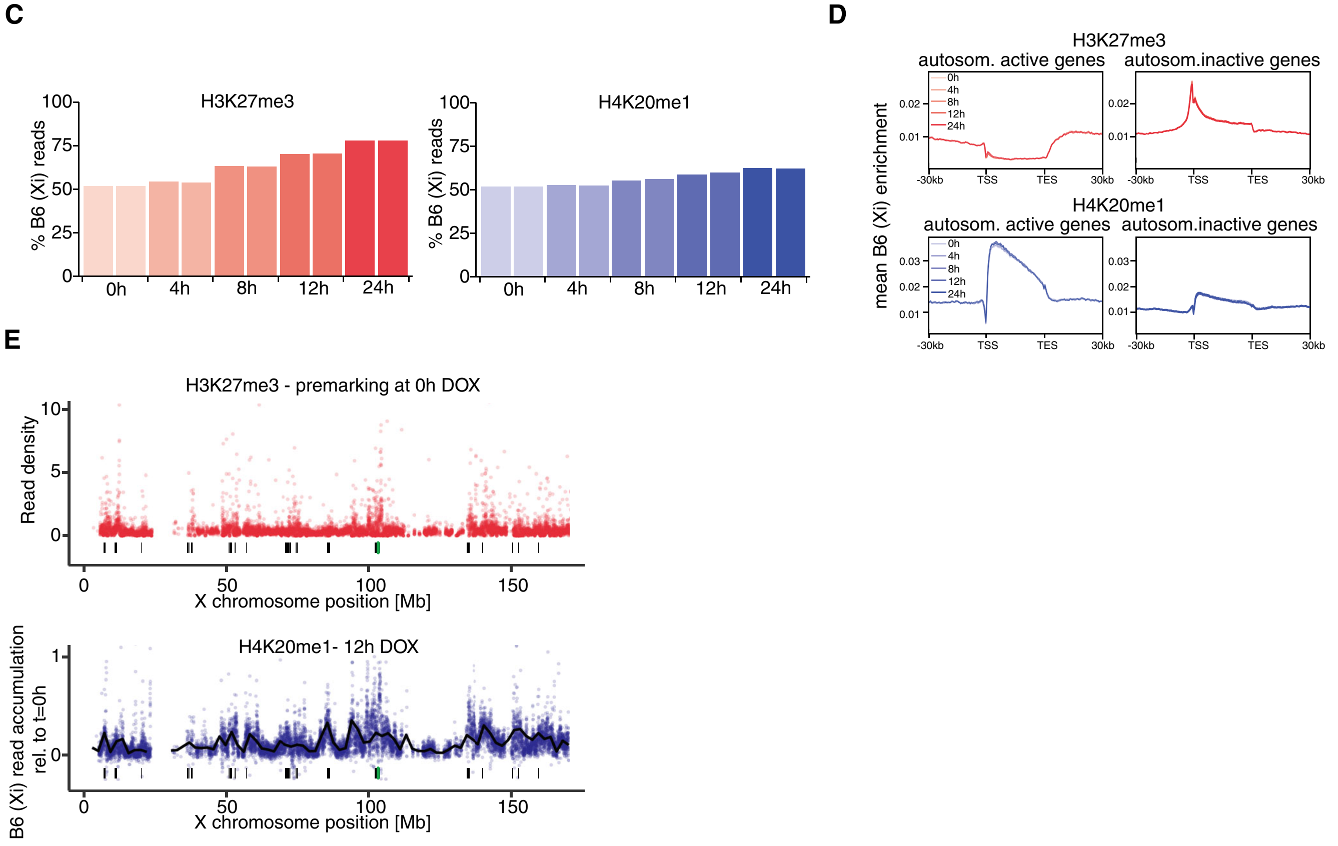

Figure EV5. H4K20me1 native ChIP-seq controls.

A Peptide array analysis of anti-H4K20me1 antibody (5E10-D8) used for native ChIP-seq. A MODified Histone Array (Active Motif) was probed with the anti-H4K20me1 antibody (5E10-D8). All peptides containing H4K20me1 were highlighted, their sequence and modifications provided in the table bellow.

B RNA FISH quantification of *Xist* induction (p510 probe) during nChIP-seq time course. At least 100 nuclei were quantified for each sample.

C Bar plot showing the percentage of B6 reads mapping to the X chromosome in the nChIP-seq time course.

D Relating to Fig 5B. Average H3K27me3 (top) and H4K20me1 (bottom) enrichment at the B6 allele over active or inactive genes ± 30 kb on autosomes. Shown is data for all time points. Note enrichment of H4K20me1 at active genes and the stability of the signal between all time points.

E Relating to Fig 4E. H4K20me1 (blue) accumulation across the Xi after 12 h of DOX treatment is compared to H3K27me3 premarking before the induction of Xist. The black line is a locally estimated scatterplot smoothing (LOESS) regression on all 10-kb windows (dots). Below each plot shown is the Xist locus (green bar) and Xist entry sites (black bars).

 © 2021 The Authors
